# Vancomycin Concentrations in Synovial Fluid Do Not Reach Chondrotoxic Thresholds After Anterior Cruciate Ligament Reconstruction With Vancomycin-Soaked Autologous Soft Tissue Grafts: An In Vivo Prospective Observational Study in Humans

**DOI:** 10.1177/03635465231169040

**Published:** 2023-05-10

**Authors:** Thomas R. Pfeiffer, Arne Althoff, Sophia Krombholz, Max Dautert, Jan-Hendrik Naendrup, Daniel Guenther, Bertil Bouillon, Mario Thevis

**Affiliations:** † Cologne Merheim Medical Center, Witten/Herdecke University, Cologne, Germany; ‡ Department of Experimental Sports Traumatology, Witten/Herdecke University, Witten, Germany; § Center for Preventive Doping Research, Institute of Biochemistry, German Sport University Cologne, Cologne, Germany; Investigation performed at the Department of Orthopaedic Surgery, Trauma Surgery and Sports Medicine, Witten/Herdecke University, Cologne, Germany

**Keywords:** anterior cruciate ligament (ACL), vancomycin soaking, vancomycin, infection, chondrotoxicity

## Abstract

**Background:**

Studies have revealed that vancomycin soaking of the anterior cruciate ligament (ACL) graft can drastically reduce the incidence of postoperative infections after ACL reconstruction. However, it remains unknown whether the chondrotoxic threshold of vancomycin in synovial fluid is exceeded during this process. Several studies investigated the chondrotoxic properties of vancomycin in in vitro experiments and described a concentration of 1000 µg/mL as the critical threshold.

**Purpose/Hypothesis:**

The purpose of the study was to measure the vancomycin concentration in synovial fluid after ACL reconstruction with vancomycin-soaked autografts. It was hypothesized that intra-articular vancomycin concentrations in the synovial fluid would not reach the chondrotoxic threshold of 1000 µg/mL after vancomycin soaking of autologous semitendinosus tendon and soft tissue quadriceps tendon grafts for ACL reconstruction.

**Study Design:**

Cohort study; Level of evidence, 3.

**Methods:**

The study enrolled 10 patients undergoing ACL reconstruction using 4-strand semitendinosus tendon autografts and 10 patients undergoing ACL reconstruction using soft tissue quadriceps tendon autografts. Before implantation, each harvested graft was intraoperatively wrapped in gauze swabs that had been soaked in a 5-mg/mL vancomycin solution. After wound closure, an aspirate of 5 mL of synovial fluid was taken from each patient. The vancomycin concentration of the aspirate was analyzed using high-performance liquid chromatography–tandem mass spectrometry. Spearman rho correlation coefficients were used to identify relationships between the parameters, and the *t* test was used to test for differences between graft types. A *P* value of <.05 was considered statistically significant.

**Results:**

The study included 20 patients (14 women and 6 men; age, 29.35 ± 11.3 years). The mean vancomycin concentration measured in the synovial fluid was 23.23 ± 21.68 µg/mL, with a minimum concentration of 2.32 µg/mL and a maximum concentration of 71.56 µg/mL. No significant difference was found between the 2 graft types (*P* = .911). Significant positive correlation (*r* = 0.644; *P* < .05) was observed only between the vancomycin concentration and the mean duration from initiation of vancomycin soaking of semitendinosus tendon grafts to implantation (13.4 ± 6 minutes). No correlations were observed between the vancomycin concentration and the duration from implantation to fluid aspiration or between the vancomycin concentration and the graft diameter (median, 8.5 mm; range, 6.0-10.0 mm) for both graft types.

**Conclusion:**

Chondrotoxic vancomycin concentrations ≥1000 µg/mL were not reached in any aspiration of synovial fluid after ACL reconstruction using soft tissue autografts that were intraoperatively soaked in a 5-mg/mL vancomycin solution. Against the backdrop of multiple studies that showed significantly reduced infection rates after ACL reconstruction when vancomycin soaking was used, this study suggests that the chondrotoxic properties of this method are negligible because of its submarginal intra-articular concentrations.

Although a rare complication, ranging from 0.14% to 4.4% in selected patient populations, postoperative septic knee arthritis after anterior cruciate ligament (ACL) reconstruction can have devastating long-term effects for an individual.^12,16,27,29^ Therefore, any effort to minimize septic knee arthritis should be undertaken. Multiple meta-analyses showed that vancomycin soaking of the ACL graft can drastically reduce the incidence of postoperative septic knee arthritis after ACL reconstruction.^2,14,17,32^

However, reservations regarding this method still prevail, and in 2021, two-thirds of the members of the prestigious ACL Study Group reported that they do not soak ACL grafts in vancomycin before implantation.^33^ The most common concerns are vancomycin-induced weakening of the graft and chondrotoxicity. Regarding weakening of the ACL graft, several in vitro studies of human tenocytes suggest that there is a safe range of vancomycin concentrations within which tenocyte viability is unaffected. Depending on the respective study, safe vancomycin concentrations varied between 2.5 mg/mL for ≤1 hour of treatment and 12.8 mg/mL for ≤6 hours of treatment.^20,31^ Likewise, vancomycin showed no effect on the biomechanical properties of ACL grafts.^11,15,26^ Several studies did not report increased rerupture rates,^3,4,7,9,21,23,25,32^ and Offerhaus et al^19^ even observed a reduced rerupture rate after vancomycin soaking.

Regarding chondrotoxicity, several in vitro studies investigated the vancomycin concentration threshold in human and animal chondrocytes and synoviocytes, finding chondrotoxic effects in concentrations as low as 1000 µg/mL.^1,18,24,34^ Because the soaked ACL graft can act as a vancomycin reservoir, the release and elution rate of human vancomycin-soaked grafts in the synovial fluid in the knee joint is of pivotal importance. Grayson et al^8^ investigated the in vitro elution rate of vancomycin-soaked animal tendons at different concentrations. The experiment showed that elution rates declined rapidly during a 1-hour interval without reaching chondrotoxic concentrations, while at the same time being above the minimum inhibitory concentration for *Staphylococcus*.

Currently, no in vivo or in vitro study has shown adverse effects with respect to vancomycin soaking. However, the preventive approach of vancomycin soaking of ACL grafts was first established in 2012, and no long-term outcome data are available.^30^ Chondrotoxic effects, especially, would become apparent only during a longer observation period. Therefore, it is pivotal to know whether this technique puts the cartilage at risk by reaching chondrotoxic thresholds. In vitro data showed that vancomycin concentrations approximate the maximum concentration within the first 10 minutes because of the rapid decrease of the elution rate.^8^ However, in vivo data within this decisive time period immediately after the implantation of vancomycin-soaked tendons are not available. Therefore, the purpose of this study was to measure the vancomycin concentration in the synovial fluid immediately after ACL reconstruction with vancomycin-soaked autografts. It was hypothesized that intra-articular vancomycin concentrations would not reach the chondrotoxic threshold of 1000 µg/mL after ACL reconstruction using vancomycin-soaked autologous semitendinosus tendon and soft tissue quadriceps tendon grafts.

## Methods

After obtaining approval from the local ethics committee (S-247/2021), we recruited patients undergoing primary isolated ACL reconstruction, with or without meniscal surgery, conducted by 1 of 2 orthopaedic surgeons (T.P. and D.G.) at our tertiary care hospital. Patients were excluded if they had sustained previous ipsilateral or contralateral ACL injuries, had undergone previous knee surgeries, or were receiving intravenous or oral vancomycin. Two groups were formed, one receiving 4-strand semitendinosus tendon grafts and the other receiving soft tissue quadriceps tendon grafts.

### Procedure of Intraoperative Vancomycin Soaking

After the quadriceps or semitendinosus tendons were harvested using respective harvesting instruments, tendons were wrapped in a vancomycin-soaked gauze compress. The vancomycin concentration was 5 mg/mL based on the standard protocol published by Vertullo et al.^30^ Subsequently, the grafts were prepared for implantation. The vancomycin was not rinsed or wiped off before implantation. A minimum soaking time of 10 minutes was ensured.

### Sample Collection Procedure

The graft was inserted under arthroscopic view. After femoral and tibial fixation, a brief period of arthroscopy was used to check for graft positioning and to remove debris. Subsequently, the remaining fluid was removed from the joint, and wound closure of the harvest site and medial portal was performed. Before suturing the anterolateral portal, the surgeon performed sterile puncture of the joint through the anterolateral portal, aiming for the knee compartment in proximity to the soaked graft in order to withdraw maximum concentrations. An aspirate of 5 mL of synovial fluid was taken from each patient. The aspirated fluid was placed in a Falcon tube and frozen at −80°C. The time intervals between initiation of vancomycin soaking and implantation and between implantation and sampling were measured.

### Analysis Technique

The vancomycin concentration of the collected synovial fluid was analyzed using high-performance liquid chromatography (HPLC) coupled with tandem mass spectrometry (MS/MS) analogous with methods previously described to determine vancomycin in serum.^5,13,28^

Samples were processed at room temperature. In brief, 100 µL of the sample was transferred to an Eppendorf tube and fortified with the internal standard (acebutolol) to a final concentration of 10 µg/mL. Proteins were precipitated by adding 400 µL of methanol, followed by thorough vortex mixing and centrifugation at 17,000*g* for 10 minutes. The clear supernatant was subsequently diluted with 0.1% formic acid in a liquid chromatography vial to a ratio of 1:100, vortex-mixed, and subjected to LC-MS/MS analysis. The samples were analyzed by reversed-phase HPLC coupled to an Orbitrap mass spectrometer (Thermo Fisher Scientific, Bremen, Germany) equipped with a heated electrospray ion source operating in positive mode. MS/MS experiments were performed, monitoring 2 diagnostic ion transitions each for vancomycin (*m*/*z* 725 → 1305; 725→1143) and the internal standard (*m*/*z* 337 → 116; 337 → 319) as quantifier and qualifier ion pairs. Estimation of the analyte concentration was achieved by means of calibration curves prepared in synovial fluid containing no vancomycin using the peak area ratios of the quantifier ion transitions of vancomycin and the internal standard. The method was validated according to the International Conference on Harmonisation criteria.^10^ Before conducting the main analysis, we conducted a preliminary test of synovial fluid after 10 ACL reconstructions without vancomycin-soaked grafts in order to validate the method by adding predetermined concentrations of vancomycin in vitro. As expected, no vancomycin was detected in synovial fluid after ACL reconstruction without vancomycin-soaked grafts.

### Statistical Analysis

Descriptive statistics were performed as follows. Mean, standard deviation, minimum, and maximum were determined for all continuous variables. The Spearman rank correlation coefficient was used to analyze correlations. A Mann-Whitney *U* test was performed to compare vancomycin concentrations between groups based on graft choice and remnant preservation. A Kolmogorov-Smirnov test was used to determine the distribution of the data, and a goodness-of-fit test including the Anderson-Darling statistic was performed to investigate whether the data were well described by the fitted distribution. To calculate the probability of a value reaching a certain threshold, the cumulative distribution function was used. Significance was set at *P* value <.05.

A post hoc power analysis was performed using G*Power Version 3.1.9.2 to calculate the power of the present study. For a *t* test comparison between 10 vancomycin concentrations after ACL reconstruction with vancomycin-soaked hamstring tendon grafts and 10 vancomycin concentrations after ACL reconstruction without vancomycin-soaked hamstring tendon grafts, a power of 0.9 was calculated. Based on the results of the Spearman rank correlation coefficient regarding the vancomycin concentrations and the duration from vancomycin soaking of semitendinosus tendon grafts to implantation, a power of 0.42 was calculated. Using the results of the independent *t* test comparing the vancomycin concentration in the synovial fluid of remnant-preserving and non–remnant preserving ACL reconstruction, an effect size of 0.97 (α = .05), and a study group of 20 patients, we calculated a power of 0.51. For a *t* test comparison between ACL reconstruction with hamstring versus quadriceps tendon grafts, a power of 0.1 was calculated.

## Results

**Figure 1.**
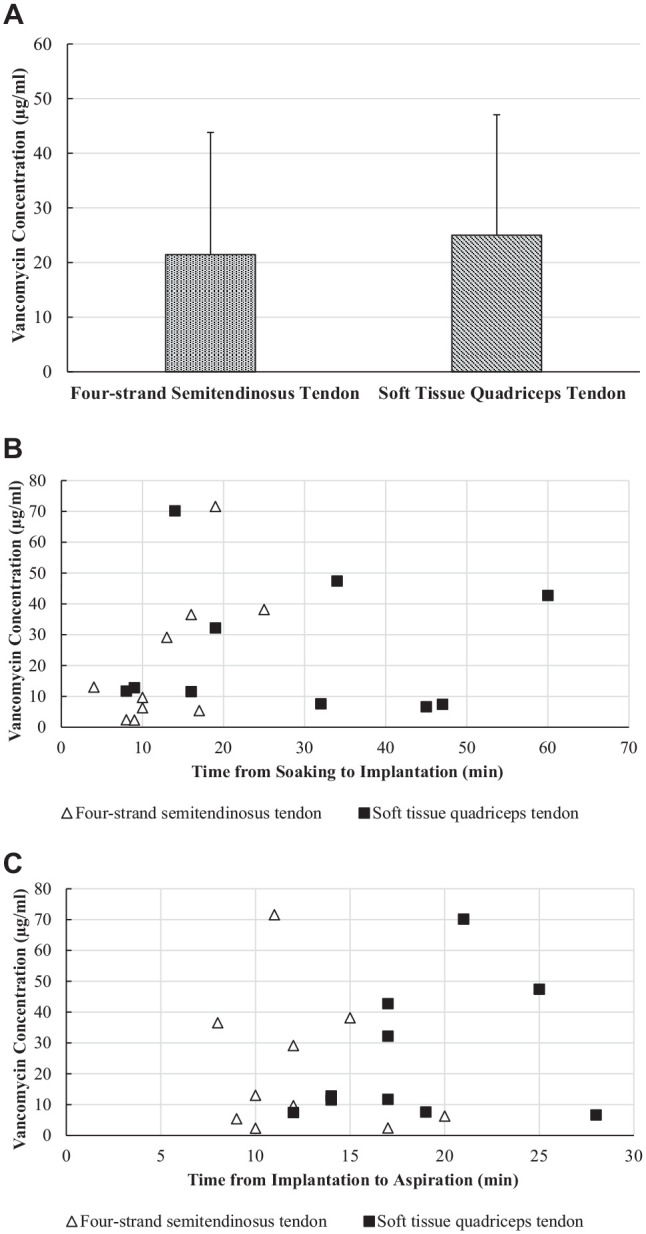
(A) Bar graph of vancomycin concentration in synovial fluid. No significant difference was seen between semitendinosus and quadriceps tendon autografts. (B) Time between soaking and implantation of the anterior cruciate ligament graft and the vancomycin concentration in synovial fluid after implantation was correlated only in semitendinosus tendon autografts (*r* = 0.644; *P* < .05). (C) No correlation was seen between time from graft implantation to sample collection and vancomycin concentration in either quadriceps tendon autografts or semitendinosus tendon grafts.

A total of 20 patients (14 women and 6 men; age, 29.35 ± 11.3 years) were included in the study. Characteristics of both groups are provided in Table 1. The mean vancomycin concentration measured in the synovial fluid was 23.23 ± 21.68 µg/mL, with a minimum concentration of 2.32 µg/mL and a maximum concentration of 71.56 µg/mL. With respect to vancomycin concentrations, no statistically significant differences were identified between semitendinosus tendon autografts (21.45 ± 22.37 µg/mL) and quadriceps tendon autografts (25.01 ± 22.02 µg/mL; *P* = .91) (Figure 1A).

**Table 1:** Descriptive Statistics^*a*^

	Quadriceps Tendon Autografts	Semitendinosus Tendon Autografts	Significance
No. of patients	10	10	
Age, y	27.0 ± 10.1	31.7 ± 12.5	NS
Sex, male/female, n	4/6	2/8	NS
Graft diameter, mm	8.7 ± 0.6	8.3 ± 0.9	NS
Remnant-preserving ACL reconstruction, n	0	4	.04
Time between vancomycin soaking and graft implantation, minutes	28.4 ± 17.9	13.10 ± 6.2	.02
Time between graft implantation and aspiration, minutes	18.4 ± 5.0	12.4 ± 3.8	.008

a Values are presented as mean ± SD unless otherwise noted. ACL, anterior cruciate ligament; NS, not significant.

The mean duration between initiation of vancomycin soaking and implantation was 20.75 ± 15.26 minutes, and the mean duration from implantation to fluid aspiration was 15.40 ± 5.33 minutes. The only significant correlation (*r* = 0.644; *P* < .05) was observed between the vancomycin concentration and the mean duration (13.4 ± 6 minutes) from initiation of vancomycin soaking of semitendinosus tendon grafts to implantation (Figure 1B). No correlations were observed between vancomycin concentration in the synovial fluid and the duration from implantation to fluid aspiration (*r* = −0.73; *P* = .841) or between the vancomycin concentration and the graft diameter (median, 8.5 mm; range, 6.0-10.0 mm; *r* = −0.026; *P* = .914) for both grafts (Figure 1C). In 4 patients, all of whom received semitendinosus tendon autografts, a substantial remnant of the original ACL was left intact, and the ACL graft was covered by the ACL stump. A significant difference (*P* = .019) was seen between the mean vancomycin concentration in the synovial fluid for remnant-preserving ACL reconstructions (7.61 ± 4.66 µg/mL) compared with ACL reconstructions without a preserved remnant (27.13 ± 22.58 µg/mL).

The vancomycin concentration was exponentially distributed. The goodness-of-fit tests showed that the data were well described by the fitted exponential distribution (Kolmogorov-Smirnov, *P* = .171; Anderson Darling statistic, 0.500). Using the cumulative distribution function, we calculated the probability of the vancomycin concentration to reach 100, 200, 500, and 1000 µg/mL. Based on the data distribution, the probability for a measurement being >100 µg/mL was 0.02; for being >200 µg/mL, it was 2.22 × 10^-18^; and for both >500 and >1000 µg/mL, it was <10^-30^.

## Discussion

The most important finding of this study was that chondrotoxic vancomycin concentrations ≥1000 µg/mL were not reached in any aspiration of synovial fluid after ACL reconstruction using soft tissue autografts that were intraoperatively soaked in a 5-mg/mL vancomycin solution. Thereby, this study adds relevant in vivo data to the ongoing discussion, suggesting that chondrotoxic effects of vancomycin soaking on the cartilage seem to be clinically insignificant and that the method constitutes a safe prophylaxis of infection in ACL surgery.

The lowest reported vancomycin concentration threshold regarding chondrotoxicity and toxicity of chondrocytes was 1000 µg/mL. The reported thresholds are based solely on in vitro studies investigating the effects of vancomycin in direct contact with chondrocytes and describing the cellular sequelae with respect to cell viability, reproduction, and necrosis. Without the extracellular and cartilage matrix that functions as an additional barrier in vivo, however, vancomycin exposure to the cells presumably results in higher cell toxicity. Therefore, the in vivo threshold for the chondrotoxicity of vancomycin concentrations might even be overestimated.^24^ However, the true chondrotoxic vancomycin concentration could be identified only by measuring vancomycin concentrations in the chondral tissue in vivo. With a mean vancomycin concentration of 23.23 µg/mL and a maximum concentration of 71.56 µg/mL in the synovial fluid on average 15 minutes after graft implantation, the present results remain distinctly below the lowest reported vancomycin concentration threshold. Importantly, all vancomycin concentrations were above the minimum inhibitory concentration of 2 µg/mL for *Staphylococcus aureus*, reported by the European Committee on Antimicrobial Susceptibility Testing.^6^ Furthermore, all but 2 measured concentrations were above the minimum inhibitory concentration of 4 µg/mL for coagulase-negative staphylococci that include clinically relevant pathogens like *Staphylococcus epidermidis*. Hence, not only did harmful concentrations seem to be ruled out, but also sufficient antibiotic concentrations were reached. The corresponding in vitro experiment by Grayson et al^8^ showed 3 major findings: (1) ACL grafts served as vancomycin reservoirs, and the graft size influenced the amount of vancomycin released; (2) the maximum elution rate occurred between 10 minutes and 1 hour, with a subsequent rapid decline; and (3) the vancomycin concentration did not exceed osteoblast and chondroblast toxicity thresholds. In contrast to this in vitro study, however, the postoperative knee joint is not a static environment. Because of postoperative hemorrhage, the intra-articular volume increases and further dilution is expected, reducing the vancomycin concentration in the synovial fluid. In a synopsis of the present in vivo and the aferomentioned in vitro study by Grayson et al, vancomycin was unlikely to accumulate in the synovial fluid because of rapid elution rates and additional dilution despite the vancomycin reservoir in the graft.

Despite their larger surface, 4-strand semitendinosus tendon grafts showed similar vancomycin concentrations compared with quadriceps tendon grafts in the present study. Regarding the relationship between vancomycin concentrations and duration of exposure, a significant correlation was identified only in semitendinosus tendons. Whether the absent tendovaginal sheath in quadriceps tendon grafts exerts an influence cannot be answered in the present study and should be investigated in vitro, especially to ensure the viability of the tenocytes. Interestingly, preservation of the remnant ACL led to significantly lower vancomycin concentrations compared with ACL reconstructions without remnant ACL, providing yet another argument for remnant preservation.

Regarding subgroup analyses, this study did not have a sufficiently large sample size to answer all research questions of interest, and the results are prone to being underpowered, as shown by the post hoc power analysis. However, even supposedly minor intraoperative interventions as in the present study always involve weighing the risk for the individual patient and the potential increase in knowledge. In addition, no meaningful a priori calculation of the sample size was possible because no reliable data were available regarding the intra-articular vancomycin concentration. Therefore, a sample size of 20 was chosen as a balance between individual risk and information gain. However, based on the calculations from the present distribution of data using the cumulative distribution function, it is very unlikely that a measured intra-articular concentration of vancomycin could be above the chondrotoxic threshold. Further, the good agreement of the fitted exponential function with the data distribution of the vancomycin concentration gives further evidence that the chosen number of cases was suitable to answer the main research question. Additionally, the time of sampling can be scrutinized. The mean duration from implantation to fluid aspiration was 15.40 minutes. Therefore, the time of sampling was just within the time interval of maximum elution as shown by Grayson et al^8^ and might miss the peak of intra-articular vancomycin concentration. Because patient safety was prioritized and fluid aspiration was reasonable only under the sterile conditions in the operating room immediately after ACL reconstruction, it was not possible to adjust the sampling time.

Although we used a single-strand graft and a multistrand graft, the results cannot be automatically transferred to bone–patellar tendon–bone grafts. Nevertheless, this study provides important in vivo data on intra-articular vancomycin concentrations after ACL reconstruction using vancomycin-soaked grafts and adds to the growing body of evidence suggesting that this is a safe method for prophylaxis of septic knee arthritis after ACL reconstruction. Even though no general recommendation for vancomycin soaking can be given because of the lack of level 1 evidence, this method has the potential to become the future gold standard. Even now it appears to be difficult to withhold this method from high-risk patients, in particular against the backdrop of multiple level 2 and 3 studies, including >20,000 patients who showed drastically reduced incidence of septic knee arthritis after ACL reconstruction.^22,32^

## Conclusion

Chondrotoxic vancomycin concentrations ≥1000 µg/mL were not reached in any aspiration of synovial fluid after ACL reconstruction using soft tissue autografts that were intraoperatively soaked in a 5-mg/mL vancomycin solution. Against the backdrop of multiple studies showing significantly reduced infection rates after ACL reconstruction when using vancomycin soaking, this study suggests that the chondrotoxic properties of this method are negligible because of its submarginal intra-articular concentrations.
